# Advances in Viroporin Function and Structure: A Comparative Analysis of Alphavirus 6K with Well-Characterized Viroporins

**DOI:** 10.3390/v17060868

**Published:** 2025-06-19

**Authors:** Vashi Negi, Andrew S. Miller, Richard J. Kuhn

**Affiliations:** 1 Department of Biological Sciences, Purdue University, West Lafayette, IN 47907, USA; vnegi@purdue.edu (V.N.); mille153@purdue.edu (A.S.M.); 2 Purdue Institute of Inflammation, Immunology, and Infectious Disease, Purdue University, West Lafayette, IN 47907, USA

**Keywords:** alphavirus 6K, alphavirus TF, IAV M2, HIV-1 Vpu, SARS-CoV-2 envelope, HCV p7, viroporins, antivirals

## Abstract

Viruses encode ion channel proteins called viroporins to assist in infection and immune evasion. The alphavirus 6K protein is classified as a member of the viroporin family of proteins. Several studies have characterized the role of 6K in alphavirus budding and infection since its discovery in the late 1970s. In this review, we summarize 6K research and discuss some unanswered questions regarding 6K biology. We highlight the similarities and differences between 6K and viroporins of clinically relevant viruses—influenza A virus, HIV-1, hepatitis C virus, and SARS-CoV-2—and address their importance as therapeutic targets. The sensitivity of these viroporins to common inhibitors and their ability to functionally complement each other underscore their potential as targets for broad-spectrum antiviral therapies.

## 1. Introduction

Viroporins are small, hydrophobic viral proteins consisting of approximately 50 to 120 amino acids. These proteins typically have three domains: a hydrophobic channel domain traversing the host cell membrane, a cytosolic domain, and a luminal domain [1]. Upon insertion into the host membrane, they oligomerize to form pores and ion channels. Viroporins contain conserved sequence motifs of basic and aromatic residues within or near hydrophobic channel domains that destabilize cellular membranes and assist in viral infection [2,3]. These common features have helped identify viroporins in various families of RNA and DNA viruses [1].

Viral life cycle events such as endosomal fusion, genome replication, particle assembly, and budding require different pH conditions and membrane modification to support infection [4,5,6]. Viroporins can remodel and conduct ions across cellular membranes via their ion channel domains to assist in different stages of the life cycle [7]. The matrix protein 2 (M2) of influenza A virus (IAV), viral protein U (Vpu) of human immunodeficiency virus-1 (HIV-1), p7 protein of hepatitis C virus (HCV), envelope (E) protein of severe acute respiratory syndrome coronavirus 2 (SARS-CoV-2), and 6K (6kDa) protein in alphaviruses play important roles during particle assembly and budding [8,9,10,11,12,13]. M2, p7, and 6K influence viral glycoprotein trafficking during infection [13,14,15]. IAV M2 also conducts protons from the acidified endosomes into the virus particle interior, reducing the pH of the viral core and triggering uncoating during entry [16].

Viroporins support viral infection by aiding viral proteins in their transportation and assembly while simultaneously impeding cellular processes and the function of cellular proteins [17,18,19]. IAV M2 inhibits autophagosome degradation by blocking fusion with lysosomes to prevent infected cells from dying [20,21]. HIV-1 Vpu, expressed in the late stages of infection, promotes cell death by inhibiting the ubiquitination of tumor suppressor protein (p53), a key player in the cellular apoptotic pathway [22]. M2 and Vpu also bind the host protein tetherin or bone marrow stromal cell antigen 2 (BST-2), an inhibitor of viral budding, to promote virus release [23,24,25].

Some viruses lack viroporins, while others may utilize larger glycoproteins or proteases to form pores and permeabilize membranes [26,27,28,29,30,31]. In viruses that encode viroporins in addition to such glycoproteins or proteases, their functions may overlap, rendering the viroporins dispensable for infection [2]. Although most viroporins are non-essential for growth, their deletion and inhibition can attenuate viruses in cell culture and in vivo. The deletion of alphavirus 6K or HIV-1 Vpu is non-lethal but severely affects particle release in infected cells [2,32,33]. Studies in animal models indicate that alphavirus and coronavirus viroporins play a crucial role in infection in vivo and act as virulence factors [34,35,36,37]. Thus, viroporins present valuable targets for the development of antivirals [36,38,39,40,41,42,43] and vaccine candidates [44,45].

## 2. Alphavirus 6K

Alphaviruses are a genus of mosquito-borne positive-strand RNA viruses that cause diseases categorized as either arthritogenic or encephalitic. In the arthritogenic group are chikungunya virus (CHIKV), Sindbis virus (SINV), Ross River virus (RRV), o’nyong-nyong virus (ONNV), Mayaro virus (MAYV), and Barmah Forest virus (BFV). These viruses cause febrile-like illness and arthritis, and severe cases can lead to chronic illness [46]. Encephalitic alphaviruses include Venezuelan, Eastern, and Western equine encephalitis viruses (VEEV, EEEV, WEEV) and have neurological disease manifestations and high mortality rates [47,48]. Both arthritogenic and encephalitic viruses are responsible for a serious health burden in areas endemic for these viruses. There are currently no vaccines or antivirals against alphaviruses except for the treatment of CHIKV [49,50]. Details of alphaviruses and their associated diseases are well reviewed [51,52,53,54].

**Figure 1:**
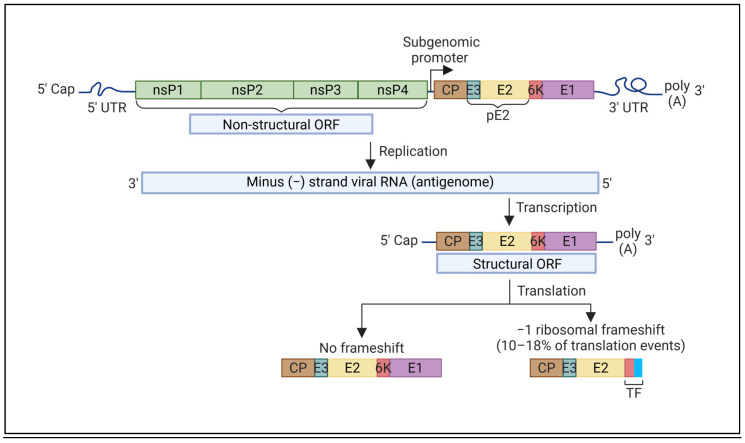
Schematic representation of the alphavirus genome and the translation of the structural polyprotein. The alphavirus genome is made up of two ORFs. At the 5′ end of the positive-sense viral RNA is the non-structural ORF, which encodes the non-structural proteins: nsP1, nsP2, nsP3, and nsP4. The structural ORF at the 3′ end encodes the structural proteins: capsid, envelope glycoproteins (pE2, E1), 6K, or TF. The structural ORF is transcribed from an internal subgenomic protomer in the minus-strand antigenome template. Occasionally, −1PRF occurs due to the presence of a conserved slippery codon (UUUUUUA motif) in the sequence of 6K, resulting in the translation of TF in place of 6K and E1. TF has the same N-terminal sequence as 6K with a unique C-terminal sequence. Abbreviations: UTR, untranslated region; nsP, non-structural protein; ORF, open reading frame; CP, capsid protein; E, envelope glycoprotein; 6K, 6kDa protein; TF, TransFrame protein; PRF, programmed ribosomal frameshifting (figure created with BioRender.com, https://www.biorender.com/, accessed on 1 June 2025).

**Figure 2:**
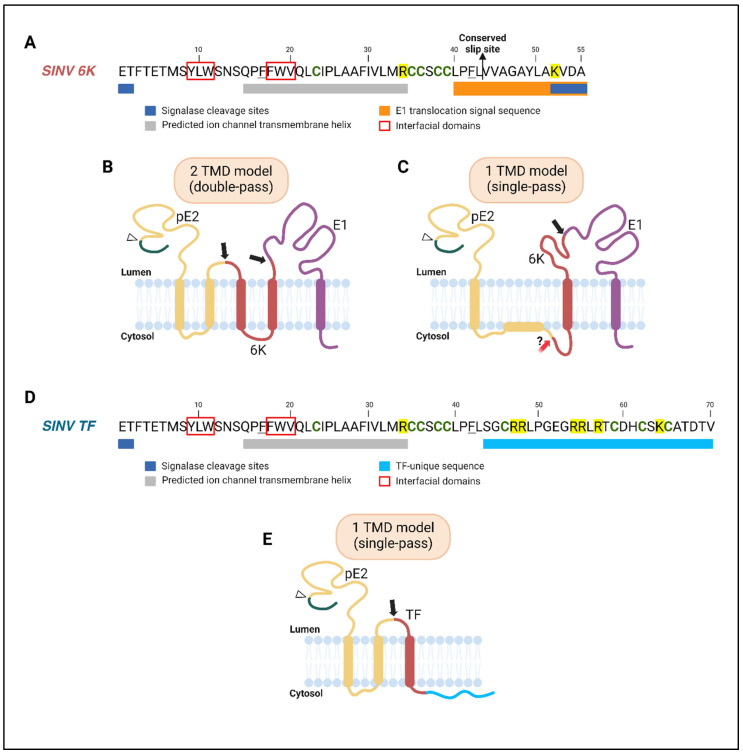
Sequences and topological models of SINV 6K and TF proteins. SINV 6K and TF represent the sequence features and topological models of alphavirus viroporins. (**A**) Predicted domains and important residues of SINV 6K are shown. Cleavage sites at the start and end of its sequence are recognized by the signalase enzyme in the ER lumen during polyprotein processing. 6K has two interfacial domains (red boxes) near the N-terminus of the predicted ion channel domain [63]. The first is in the pre-transmembrane region, while the second may be a part of the ion channel TMD occupying the amphipathic zone of cellular membranes. Aromatic residues outside these interfacial domains, which may be involved in membrane interaction, are underlined in black. Cysteine residues are emboldened in green, and basic residues are highlighted in yellow. Cysteines can undergo post-translational modifications or form disulfide bonds. The conserved basic residue R34 is important for channel activity [35]. The conserved slip site where −1 PRF occurs is indicated with a black arrow. The E1 translocation signal at the 6K C-terminus is shown in orange. (**B**) The two-TMD model of SINV 6K is shown. 6K N- and C-termini are in the ER lumen and accessible by signalase enzyme (black arrows) for cleavage from pE2 and E1. 6K has two transmembrane helices: the first is involved in the formation of an oligomeric ion channel [63,64], while the second is needed for E1 translocation [60]. The two helices are separated by a short cytoplasmic loop containing some or all of the four cysteine residues—C35, 36, 38, and 39—which are not palmitoylated [65]. If these residues are present in the ER membrane, they are no longer accessible for palmitoylation. This scenario may place the R34 residue in the hydrophobic region of the ER membrane, leading to membrane destabilization. The furin cleavage site in pE2 that yields E3 and E2 is shown with a white arrowhead. (**C**) The one-TMD model of SINV 6K is depicted [66]. 6K has a single TMD with the N-terminus in the cytosol and the C-terminus in the ER lumen. This occurs as E2 TM2 does not span the ER membrane but is bound at the cytosolic interface. Cysteine residues in 6K are inaccessible to the cytosolic palmitoylation machinery. 6K is cleaved from pE2 by an unknown protease (red arrow). (**D**) Predicted domains and important residues in the sequence of SINV TF viroporin are shown. TF has a unique C-terminal sequence (blue) containing five additional cysteines (emboldened in green) and basic residues (highlighted in yellow). (**E**) TF has a single TMD with the N-terminus in the ER lumen and the C-terminus in the cytosol. In approximately 20% of translation events, E2 TM2 undergoes membrane integration and stimulates −1 PRF to produce TF. Cysteine residues in TF at the C-terminus of the TMD are accessible for palmitoylation in the cytosol. For sequence alignment of 6K and TF proteins from different alphaviruses, refer to the review by Ramsey and Mukhopadhyay (2017) [65]. Abbreviations: SINV, Sindbis virus; 6K, 6kDa protein; E, envelope glycoprotein; TMD, transmembrane domain; TF, TransFrame protein; ER, endoplasmic reticulum; C, cysteine; PRF, programmed ribosomal frameshifting (figure created with BioRender.com https://www.biorender.com/, accessed on 1 June 2025).

An alphavirus particle consists of 240 copies each of the envelope glycoproteins E1 and E2, embedded in a host cell-derived lipid bilayer, which form 80 trimeric spikes. Each spike is a trimer of E1–E2 heterodimers in a 1:1 ratio. The glycoproteins interact with entry receptors to initiate infection. Underneath the lipid bilayer lies the nucleocapsid core, made up of 240 copies of the capsid protein (CP) and the viral genome. An additional envelope glycoprotein, E3, may also be present in some viruses but is generally cleaved from precursor protein pE2 during virus maturation and is not incorporated into the budding virion [55]. The proteins CP, pE2, and E1 are encoded as part of the structural polyprotein, along with the viroporin 6K (Figure 1). 6K was one of the first viroporins identified, alongside IAV M2 and poliovirus 2A and 3A proteins [56,57,58]. 6K acts as the translocation signal for E1, preceding E1 in the polyprotein sequence (Figure 2A) [59,60,61]. pE2, 6K, and E1 are cleaved from each other by the cellular signalase enzyme [62].

In 2008, a highly conserved ribosomal frameshift site was discovered in the N-terminal sequence of 6K [67]. Programmed ribosomal frameshifting (PRF) results in the translation of the TransFrame protein (TF) in place of 6K and E1 (Figure 1). Both 6K and TF are synthesized as part of the structural polyprotein and are co-translationally integrated into the host cell endoplasmic reticulum (ER) membranes. TF protein is produced with a frameshift frequency of 10–18% in SINV and Semliki Forest virus (SFV), and between 5 and 40% in other alphaviruses [67]. TF and 6K share the same N-terminal sequence, but TF has a unique and typically longer C-terminal sequence (Figure 1 and Figure 2). Upon polyprotein processing, 6K and TF together are in equimolar amounts relative to pE2 in infected cells, but are present in sub-stoichiometric ratios in the virus particle [68,69,70]. The degree of post-translational modifications on the cysteine residues of 6K and TF also differs, resulting in differences in their molecular size and acylation patterns [65,67]. These proteins can undergo palmitoylation which adds a 16-C (carbon) chain to cysteine residues, increasing their hydrophobicity and localization to the plasma membrane (PM) [71]. Shared cysteine residues are palmitoylated in SINV TF but not in SINV 6K (Figure 2), potentially contributing to differences in their cellular localization and function [71]. The discovery of TF prompted a reexamination of prior studies on 6K to delineate the individual roles of 6K and TF [65].

### 2.1. Relative Abundance of 6K and TF

Before TF was discovered, studies conducted on SINV and SFV showed that the relative abundance of 6K in cell lysates is similar to glycoproteins but considerably lower in purified particles. Through the use of pulse-chase labeling and detection with 6K antibodies, the abundance of SINV 6K was found to be 20–30 copies per virion, or 8–12.5% relative to E1–E2 heterodimers [68,70,72]. These figures were later adjusted for the difference in the number of cysteine residues in TF and 6K (Figure 2A,D), and the relative abundance of SINV TF in the virions was estimated to be 4.4–6.9% relative to E1–E2 heterodimers [67]. Another study used densitometry to suggest that the number of 6K copies is half of TF in purified SINV virions [35]. In SFV virions, the amount of TF is approximately 15% of the capsid protein, while copies of 6K are less than 25% of TF [67]. The authors of the study argued that since the amount of TF in the virion and the 10–18% frameshift efficiency are similar, the inclusion of TF in the virion is likely not accidental [67].

6K and TF have not been identified in cryo-EM reconstructions of alphaviruses due to their sub-stoichiometric arrangement, and structural information for either protein is not available yet. While some studies have investigated the function of TF during infection, its role as a part of the virion remains unclear. The current consensus is that 6K is mainly retained in infected cells while TF is predominant in budding virus particles [65].

### 2.2. Membrane Topology and Orientation of 6K and TF

An initial topology model predicted that upon translation, alphavirus 6K spans the ER membrane twice with its N- and C-termini in the lumen (Figure 2B) [60,70,73]. After signalase cleavage, the C-terminus of 6K may translocate to the cytosol, similarly to E2 glycoprotein [65,74,75,76]. This model is consistent with the observation that purified 6K proteins expressed in planar lipid bilayers have a single transmembrane domain (TMD) [64]. When TF is translated, its unique C-terminal tail is exposed to the palmitoylation machinery in the cytosol (Figure 2E) [71]. However, this model does not explain why the shared cysteine residues at the end of the first TMD, upstream of the slip site, are palmitoylated in TF but not in 6K, despite being oriented toward the cytosol [71].

A new model proposed by Harrington et al. (2020) suggests that SINV 6K and TF share a single TMD with inverted topologies in the ER (Figure 2C,E) [66]. This was shown using a glycosylation-based translocation assay in membrane vesicles, a glycosylatable fluorescence reporter system in cell culture, and computational analysis. During translation, the second TMD of E2 (E2 TM2) remains largely at the cytosolic interface of the ER membrane due to its marginal hydrophobicity. As a result, SINV 6K spans the ER membrane once with an N_cytosol_–C_lumen_ orientation, and its cysteine residues exposed to the ER lumen are unavailable for palmitoylation (Figure 2C). Occasionally E2 TM2 undergoes membrane integration with approximately 20% efficiency and stimulates −1 PRF. This leads to the formation of SINV TF with an N_lumen_–C_cytosol_ orientation (Figure 2E). The shared cysteine residues in TF are now exposed to the cytosol for palmitoylation. Harrington et al. (2020) also suggest that this −1PRF mechanism is conserved amongst alphaviruses, as the predicted frameshift efficiencies are similar to the predicted probabilities of E2 TM2 membrane integration, generating the necessary force for PRF [66,67,77].

Under the new model, 6K and TF could form distinct ion channels with different functions, assuming that these channels are selective and display directionality [64,78]. With 6K N-terminus in the cytosol, it is unclear how 6K and pE2 are proteolytically cleaved as the cleavage site is no longer accessible to the signal peptidase enzyme (Figure 2C) [60]. In the absence of structural data, a consensus on the orientation of 6K and TF in intracellular membranes is also lacking. However, it is generally believed that TF, but not 6K, is palmitoylated, and mutations or deletions in the palmitoylation sites result in defective proteolytic processing and budding [35,61,71,72,79,80]. A comprehensive list of defects associated with deletion or mutation in 6K and TF is provided in Table 1.

### 2.3. Pore-Formation and Ion Channel Activity of 6K and TF

The alphavirus 6K protein has ion channel activity that affects viral budding and particle release [81,82]. Its pore-forming ability was first demonstrated in SFV, where it permeabilized membranes in an *E. coli* cell lysis assay [58]. 6K proteins from RRV and BFV, alphaviruses endemic to Australia, conduct cations when expressed in planar lipid bilayers [64]. These studies gave rise to the theory that 6K functions as a viroporin to assist in infection.

Further insight into 6K function emerged when two conserved interfacial domains were identified at the start of the first predicted TM helix in SINV 6K (Figure 2A) [63]. Mutating these domain residues to alanine reduced the toxicity and membrane permeabilization ability of the protein when expressed in *E. coli* cells. Thus, 6K’s ion channel activity was attributed to its N-terminal TM helix [13]. CHIKV 6K conducts ions in lipid bilayers and is inhibited by the IAV M2 channel inhibitor amantadine [88]. SINV 6K is also sensitive to the HIV-1 Vpu channel inhibitor, 5-N, N-Hexamethylene amiloride (HMA), which hampers SINV infection [13]. These studies further support 6K’s viroporin activity and sensitivity to channel inhibitors.

Since 6K and TF share the TM helix associated with 6K’s channel activity (Figure 2), TF may also function as an ion channel. An *E. coli* growth assay showed that the overexpression of SINV TF is toxic to bacterial cells, whereas SINV capsid protein is not, suggesting that TF retains the pore-forming ability of 6K [35]. During infection, TF assists in alphavirus budding and acts as a virulence factor [65]. The deletion of SINV TF attenuates the virus and leads to better survival outcomes in infected mice [35]. A recent electrophysiology study showed that CHIKV TF can also conduct ions [89]. So far, studies have not assessed the ion conductance of 6K or TF in the context of infection or through overexpression in mammalian cells. To attribute TF’s function as a virulence factor to its ion channel activity, experiments assessing TF’s role during infection, independent of 6K, are needed. This is challenging as there is currently no tool to generate a TF-only virus with the structural proteins in their correct orientations due to reasons discussed in a past review [65].

### 2.4. Intracellular Localization and Function of 6K and TF

**Figure 3:**
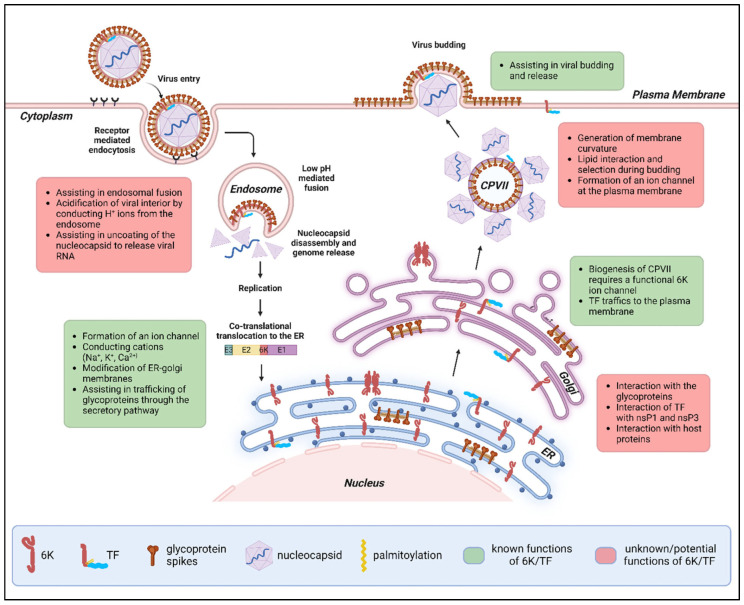
Known and potential roles of 6K and TF proteins during the alphavirus life cycle. 6K and TF are expressed in the internal membranes of the ER and the Golgi apparatus. TF localizes to the plasma membrane and gets incorporated into the budding virion. Based on the existing literature, the known (green boxes) and potential (red boxes) roles of these proteins are highlighted alongside their intracellular locations. Abbreviations: 6K, 6kDa protein; TF, TransFrame protein; CPVII, cytopathic vacuole II; ER, endoplasmic reticulum; nsP, non-structural protein (figure created with BioRender.com, https://biorender.com (accessed on 1 June 2025).

Localization assays conducted in RRV- and SINV-infected cells provide strong evidence that 6K is mainly ER-resident (Figure 3) [13,37]. Using a rabbit polyclonal antibody raised against RRV 6K, Taylor et al. (2016) showed that 6K colocalizes with the ER marker calnexin while RRV E2 is found both in internal membranes and at the PM [37]. 6K did not colocalize with markers for the Golgi, early endosomes, lysosomes, or autophagosomes that accumulate in late-stage infection. Elmasri et al. (2022) used a miniSOG-tagged SINV 6K virus to track its localization [13]. In the absence of TF, 6K was mainly found in the ER, Golgi, and dynamic vesicles of the late secretory pathway but was absent at the PM [13]. 6K colocalizes in internal membranes with mCherry-tagged E2 protein [13]. Another study analyzed the ability of CHIKV 6K to permeabilize different cellular compartments and found that 6K disrupts liposomes mimicking the ER more than the PM [88]. Cholesterol-rich liposomes mimicking the PM are more rigid compared to the ER liposomes [88,90]. It has also been argued that the ER membrane is slightly thinner than the PM [91]. The lipid composition of a membrane and its thickness may influence the ability of a lipophilic protein like 6K to form functional channels [81,88]. These factors, in addition to the lack of palmitoylation and the presence of potential binding partners, may affect its intracellular localization in infected cells.

The palmitoylated TF protein is expected to translocate to the PM at the site of alphavirus budding, to be incorporated into the budding virus particle, although this has not been demonstrated experimentally (Figure 3) [65]. However, when expressed alone in mammalian cells, SINV TF protein is retained in the ER [35].

#### 2.4.1. Endoplasmic Reticulum (ER)

##### 6K Is Needed for Efficient E1 Translocation and Polyprotein Processing

Early studies in SFV showed that the 6K C-terminus has the translocation signal for E1 [60]. When 6K is deleted, the translocation signal in the C-terminus of pE2 functions as the signal sequence for E1. Thus, the ∆6K SFV mutant is viable, but translocation and cleavage are less efficient, which could explain the decrease in titer [60]. Mutations around the signal peptidase cleavage sites at the C-terminus of pE2 and 6K result in altered proteolytic processing, affecting particle release from infected cells [61]. A mutation in the SINV 6K sequence near the cleavage site, K52A, that altered 6K-E1 cleavage led to the formation of viral particle aggregates on the cell surface [61].

##### The 6K Ion Channel Permeabilizes Membranes to Conduct Cations

The expression of 6K and TF in *E. coli* cells leads to increased membrane permeabilization and toxicity, likely due to pore formation [35,58]. RRV and BFV 6K proteins preferentially conduct cations in planar lipid bilayers in the order Na^+^ > K^+^ > Ca^2+^ >> Cl^−^, with a 15–16-fold preference for Na^+^ over Cl^−^ and a 3–6-fold preference for monovalent Na^+^ over divalent Ca^2+^ [64]. Changes in the concentration of monovalent ions during infection can affect viral entry, host protein synthesis shut-off, assembly, and release [92,93,94]. This is a general feature of viral infections and is likely modulated by the presence of viroporins [7]. Thus, 6K and, perhaps, TF support different aspects of viral infection by conducting cations, although the specific mechanisms are not well understood.

While most viroporins are selective for protons and monovalent cations, a few, such as picornavirus 2B and rotavirus NSP4, conduct calcium ions to disrupt host signaling pathways, induce apoptosis, and activate inflammasomes [3]. SINV 6K expression in *Xenopus* oocytes raises calcium ion levels, leading to cell shrinkage and cell death, although this effect could not be attributed to 6K-specific channel activity [78]. 6K may be involved in disrupting calcium homeostasis either by directly leaking calcium ions from the ER into the cytoplasm through its channel or by indirectly activating endogenous ER channels [1,3,78]. Further investigation of 6K ion channel activity during infection in mammalian cells is needed to confirm whether 6K conducts calcium ions to assist in infection.

##### 6K/TF May Interact with Other Viral Proteins in the ER

The ER affords proximity to structural proteins during and after proteolytic processing. An intraviral interactome generated for CHIKV using the co-immunoprecipitation of tagged CHIKV proteins suggests that 6K and TF interact with the glycoproteins [95]. The study also suggests that TF interacts with nspP1 and nsP3 in a palmitoylation-dependent manner. Whether this interaction occurs in the ER or elsewhere is not known, since whole-cell lysates were used in the study.

In a pulse-chase labeling experiment, monoclonal antibodies against the E1–E2 heterodimer captured 6K from SFV-infected baby hamster kidney (BHK) and chicken embryo fibroblast (CEF) cells, while anti-capsid antibody did not [69]. This was seen after a short 10 min chase, suggesting that 6K is associated with pE2 and E1 immediately after protein synthesis in the ER. The amount of 6K captured by the antibody is similar to the total amount of 6K in the 10 min chase sample. This association is also observed in 30, 90, and 180 min chase periods, with a diminishing pE2 signal, indicating possible 6K interaction with spikes beyond the ER. Through the use of an SFV clone deficient in E1, 6K and pE2 are again found to co-immunoprecipitate, suggesting that 6K-pE2 interaction does not require E1 protein [69]. Only one form of the 6K protein was detected in this study, and it is uncertain whether it was 6K or TF. Interaction with either protein might facilitate glycoprotein trafficking from the ER to the site of budding. This is further supported by studies where the mutation or deletion of 6K affects either the amount of E2 expression on the PM or the ability of E1 and E2 to associate with each other to form spikes (Table 1) [13,70,72,80,83].

#### 2.4.2. Golgi Compartment

##### 6K Is Involved in Glycoprotein Trafficking and the Biogenesis of Cytopathic Vacuoles Type II (CPV-IIs)

SINV 6K deletion affects glycoprotein transport through the secretory pathway and disrupts the formation of CPVIIs, which are thought to be budded vesicles originating in the Golgi [13]. CPV-IIs, coated with nucleocapsid cores on their membranes and E1–E2 glycoproteins in their interior, transport viral structural components to the PM, although this model of alphavirus assembly is not confirmed [96,97]. The insertion of a 15-amino-acid sequence at position 29 in the predicted TM1 helix of SINV 6K leads to a slower rate of pE2 to E2 conversion, suggesting that 6K mutation affects either the transport of pE2 or its accessibility to the furin enzyme in the Golgi [72].

#### 2.4.3. Plasma Membrane (PM)

##### 6K/TF Modifies Membrane Curvature and Affects Particle Morphology

While 6K is primarily ER-resident, TF is believed to traffic to the PM and assist in budding. Some known TF-specific properties and functions during alphavirus infection are listed in Table 2. Studies conducted prior to the discovery of TF show that SINV 6K mutations lead to the formation of multicored particles and an increased number of cell-associated viruses compared to wild-type virus [61,72,79,98]. Loewy et al. (1995) observed that SFV 6K deletion leads to an increase in the accumulation of intact nucleocapsid cores on the inner side of the PM in electron microscopy images of infected BHK cells [81]. Gaedigk-Nitschko et al. (1991) suggested that cysteine palmitoylation allows SINV TF (originally identified as 6K) to bend the PM and assist in the interaction between the nucleocapsid cores and E1–E2 trimers during assembly [61]. TF might also be involved in lipid flipping and asymmetry, further assisting in budding [61]. Taken together, these studies suggest that TF modifies PM curvature to promote efficient alphavirus assembly and budding, but further research is needed to confirm this.

##### The 6K/TF Ion Channel Permeabilizes the PM During Budding

A functional ion channel is essential for efficient alphavirus budding [13]. The loss of SINV 6K channel activity greatly reduces viral titer but can be partially rescued by IAV M2, HIV-1 Vpu, and HCV p7 channels [13]. The wild-type 6K protein and its channel chimeras are absent from the PM in infected BHK cells, suggesting that a functional channel can promote budding without being present at the PM. These chimeric viruses lack TF, and the study did not assess the role of TF channel activity in particle release, but earlier studies provide valuable clues.

Mutations in the pre-transmembrane interfacial domains of SINV 6K/TF can lead to budding defects [63]. The mutant particles retain wild-type morphology but are unable to detach from the PM of infected BHK cells based on electron microscopy images [63]. The interfacial domains are essential for membrane permeabilization but not for membrane integration [63]. Thus, these domains likely affect the formation of a functional channel or pore, which, when mutated, impairs the pinching off of virus particles from the PM. A model proposed by Sanz et al. (2005), prior to TF discovery, suggests that 6K pores dissipate membrane potential around budding sites to provide the energy necessary for particle release [102]. SINV budding is greatly inhibited by low-ionic-strength (LIS) media, and the defect is reversed by providing normal media or introducing specific mutations in the E2 glycoprotein [103,104]. Thus, investigating whether TF conducts ions and modifies membrane curvature at the PM can help in understanding the mechanism of TF’s function in alphavirus budding.

##### 6K/TF Affects the Thermostability of the Virus and Assists in Budding

SFV lacking 6K is more temperature-sensitive than wild-type SFV, yielding a relative titer of 30% at 30 °C and of 10–20% at 40 °C in chicken embryo fibroblasts (CEFs) [81]. A 6K deletion has a minimal effect on viral titer in insect cells, where budding can occur internally, compared to mammalian cells [81,105]. These results suggest that 6K and TF could provide a growth advantage to the virus in birds, a natural alphavirus host with a high internal body temperature of 40–41 °C, and support virus release at the PM in mammalian cells, where budding from internal membranes does not occur.

##### 6K/TF May Interact with Lipids to Promote Viral Budding

The interaction of viral proteins with cholesterol facilitates the assembly and budding of enveloped viruses at the PM, as demonstrated in IAV infection [106]. Cholesterol is required for alphavirus exit, but its interaction with viral components during assembly remains unclear [107]. Released viral particles may have distinct lipid compositions compared to host cell membranes [108,109]. The SINV viral membrane is rich in cholesterol and sphingomyelin [108]. Viroporins can assist in viral budding in a cholesterol-dependent manner. IAV M2 has a cholesterol-binding site that allows it to interact with lipids and aid in budding [106,110]. Whether 6K and TF exhibit a similar association with cholesterol in infected cells is unknown [88]. So far, there is no evidence of their interaction with any lipid moieties. On the contrary, conflicting reports exist that question the importance of cholesterol during budding. Studies in SFV show that the lipid composition of the virus particles released from BHK cells resembles the host cell PM [111,112]. Hafer et al. (2009) argue that the cholesterol dependence reported in previous studies was an experimental artifact and that high SINV titers can be obtained from delipidated insect cells [113]. Notwithstanding, lipid selection during assembly would not be unique to alphaviruses and investigating 6K or TF’s role in this process is a potential avenue for future research [109,114].

##### 6K/TF May Interact with E2 During Budding

Both 6K and TF can co-immunoprecipitate with E2, and mutations in the proteins are associated with altered particle morphology [61,69,81,95]. Based on these studies, 6K is hypothesized to act as a spacer during assembly. This model suggests that the binding of 6K or TF to E2 facilitates the interaction between the cytoplasmic tail of E2 and the capsid protein during assembly [65]. The model is further supported by the observation that *trans* complementation does not rescue budding defects caused by the partial deletion of SINV 6K, as proximity to the glycoproteins might be necessary for efficient assembly and egress [80].

#### 2.4.4. In the Virus Particle

The amount of 6K and TF present in the virus particle varies between different virus species and is less than the number of glycoprotein copies. Pulse-chase experiments and gel analysis of purified virions show that TF is more abundant than 6K [67,68]. Much of the TF protein produced in cells is incorporated into budding viruses [67]. The C-terminus of TF also regulates its palmitoylation, affecting PM localization, particle morphology, and TF’s ability to antagonize host interferon responses [71,99,101]. Mutations in cysteine residues in 6K and TF do not hamper growth in cell culture but affect the morphology of the budding virus [71]. Without structural data, it is not known whether 6K and TF are present as oligomeric channels or as monomers in the icosahedral particle. The role of these proteins in the virus particle is poorly understood, leaving several questions unanswered.

##### Does the Alphavirus Particle Contain an Ion Channel? 

If membrane leakiness is detected during viral entry, it is exclusively due to virion components since gene expression is yet to occur. Viroporins are generally in very low amounts in virus particles and are not surface-exposed. Since other pore-forming proteins, mainly glycoproteins for enveloped viruses and capsids for non-enveloped viruses, are accessible, it is generally believed that viroporins are dispensable for viral entry [2]. The IAV M2 channel is an exception, as it conducts protons to initiate uncoating and genome release. M2 function is critical for entry, and its inhibition perturbs infection [1,39,115]. Alphaviruses are sensitive to changes in pH, and a low-pH environment is needed for endosomal fusion [116,117,118]. Low-pH treatment can lead to the shrinking of the nucleocapsid, as reported in a 1975 study for SFV at pH 5.6–6.4, although no effect was observed in SINV cores in a subsequent 1991 study [51,119,120]. The presence of a 6K or TF ion channel in the particle mediating entry is not essential, as the deletion of 6K in SINV does not greatly influence the specific infectivity of the virus [13,35] but may facilitate the subsequent disassembly of capsid and glycoprotein components.

##### Does 6K or TF Affect E1 Trimerization and Fusion During Entry? 

In a study by McInerney et al. (2004), SFV 6K deletion did not affect E1 trimerization kinetics but reduced low-pH-mediated liposomal fusion by one-third [82]. However, when the 6K-deficient SFV particles were allowed to attach to the liposomes prior to low-pH treatment, fusion increased by 61% compared to the sample without prior attachment. The authors reasoned that the loss of 6K accelerates the transition of virus particles from a fusion-active state to a fusion-inactive state once the pH is lowered. It is known that once E1 trimerization occurs at low pH in the absence of a membrane, the virus is no longer capable of fusion [121,122]. E1 trimerization is also enhanced by the presence of cholesterol in the membranes [123,124]. The authors speculated that the absence of 6K alters the E1–E2 spike conformation, making them more sensitive to low-pH inactivation. They proposed that E1 trimerization occurs after membrane association and is promoted by the interaction of E1 with specific lipids like cholesterol and sphingolipids. In the absence of 6K or TF, E1 trimerization may occur without association with a cholesterol- and sphingolipid-rich membrane, leading to the formation of E1 fusion-inactive trimers. This supports the idea that 6K and TF may be involved in lipid selection.

## 3. Comparison to Other Viroporins

In a recent study, the loss of the SINV 6K channel was partially rescued by viroporins of diverse virus families IAV, HIV-1, and HCV, suggesting analogous functions [13]. IAV, an enveloped virus with a negative-stranded segmented genome belonging to the *Orthomyxoviridae* family, encodes the M2 viroporin which plays a role in viral entry and exit and was the first known antiviral target in the viroporin family [1,40]. HIV-1, the causative agent of acquired immunodeficiency syndrome (AIDS) and a retrovirus, encodes Vpu, an ion channel protein involved in viral budding and antagonizing host immune responses [9,125]. HCV, an enveloped single-stranded positive-sense RNA virus of the genus *Hepacivirus* in the *Flaviviridae* family, encodes the p7 viroporin. p7 is considered a non-structural protein since it is not incorporated into the budding virion [126]. Coronavirus E viroporin is a promising antiviral target involved in viral pathogenesis and is highly conserved between SARS-CoV and SARS-CoV-2 variants of concern [36,127]. Despite lacking overall sequence homology, these proteins can support the infection processes of unrelated viruses in a manner similar to the native viroporins. The common features that might enable this complementation can serve as a foundation for the development of broad-spectrum antivirals against these viroporins.

### 3.1. Functional Complementation Studies with 6K, M2, Vpu, p7, and E

HIV-1 Vpu can partially complement SINV 6K function when introduced in trans to compensate for the loss of titer and cytopathy in infected cells by rescuing defects in polyprotein processing and membrane permeability [85]. In contrast, 6K itself is unable to rescue defects in the same SINV 6K deletion mutant when introduced in trans [80]. Another study showed that the Vpu TMD can partially rescue defects associated with the loss of the SINV 6K channel such as glycoprotein trafficking, CPV-II formation, and budding in a cis-complementation assay [13]. HCV p7 and IAV M2 are less complementary to 6K than Vpu [13]. It is important to note that these chimeras lack the TF protein and were generated under the assumption that 6K has two TMDs with its N- and C-termini in the ER lumen, which would limit the extent of complementation. When the single-TMD topology of 6K is taken into account, E1 in the SINV-p7 chimera would be in the wrong orientation in over 85% of translation events [66]. This could explain why p7 performs worse than Vpu in complementing the function of SINV 6K.

Studies that replace viroporins of other viruses with M2 can offer insights into the functions and antiviral potential of poorly characterized viroporins. The ion channel domain of Vpu in Simian Human Immunodeficiency Virus, closely related to HIV-1, can be replaced with that of IAV M2, resulting in a rimantadine-sensitive chimeric virus [128]. The chimera behaves similarly to the wild-type virus with slightly delayed growth kinetics, and the chimeric Vpu-M2 channel shares the subcellular localization pattern of wild-type Vpu. M2 can compensate for channel-inactivating mutations in HCV p7 when expressed in trans and assist in HCV infection [15]. Co-expression with M2 also makes the amantadine-resistant genotype 2a HCV strain partially sensitive to amantadine [15]. Conversely, the p7 channel can replace the function of M2 in a cell-based assay [129]. Unlike the cis-complementation of SINV 6K by Vpu, the complete deletion of HCV p7 cannot be compensated for by M2 or Vpu [130]. Recent data suggests that SARS-CoV-2 E protein and SINV 6K may also play complementary roles, as E is able to rescue defects due to the loss of 6K channel function [131].

Functional complementation studies provide clues about the shared functions of viroporins that can be targeted with common drugs. To understand what dictates the ability of these proteins to complement each other, it is important to understand the functions of these viroporins in the context of their respective viruses. Structural features, common sequence motifs, and channel organization further inform functional complementarity.

### 3.2. Subcellular Localization of 6K, M2, Vpu, p7, and E

M2, Vpu, p7, and E are mainly expressed in membranes of the ER and the trans-Golgi network (TGN) similarly to 6K [1,11,132,133,134,135]. M2, Vpu, and TF are also expressed on the PMs to assist in viral egress since influenza viruses, retroviruses, and alphaviruses bud at the PM [1]. Copies of IAV M2 present in the incoming virus particle are involved in entry [132]. HIV-1 Vpu can also localize to the endosomes, where it disrupts the trafficking of a cellular protein, tetherin [133]. Although most HCV proteins are mainly ER-resident, supporting viral assembly and budding, a fraction of ectopically expressed p7 protein localizes to the PM and the mitochondria [129,134,136]. Coronavirus E protein is mainly involved in viral assembly and budding at the ER–Golgi intermediate compartment (ERGIC) [137,138]. It is unclear whether E can localize to the mitochondria, although the expression of E protein has been shown to interfere with ER–mitochondrial contact sites [139].

### 3.3. Ion Selectivity of 6K, M2, Vpu, p7, and E

Viroporins generally lack the high ion selectivity and gating mechanisms characteristic of classical ion channels. Not all viroporins may function as bona fide ion channels. Defining their ion channel activity typically requires the demonstration of (i) measurable membrane conductance (e.g., in oocytes), (ii) single-channel currents, (iii) responsiveness to ion channel blockers, (iv) ion selectivity, and (v) mutational effects on channel function. Their channel activity may also be influenced by factors such as membrane composition, ion concentration, and pH. Most viroporins have a preference for cations over anions, albeit minimally. Their ability to disrupt host cell membranes and conduct ions helps the respective viruses during various life cycle stages.

IAV M2 can conduct monovalent cations H^+^, Na^+^, and K^+^ [3,56]. Electrophysiology studies conducted in whole-cell systems as well as liposomes show that IAV M2 conducts protons with its low-pH-activated ion channel [140,141,142]. HIV-1 Vpu and HCV p7 do not have a preference for a single ion but generally conduct monovalent cations Na^+^ and K^+^ over anions [9,143,144,145]. Additional reports on p7 ion channel activity show that p7 can conduct protons to support HCV infection and has a preference for Ca^++^ over K^+^ ions [15,42]. SARS-CoV E protein is also mildly cation-selective and can conduct Na^+^, K^+^, H^+^, and Ca^++^ ions [146,147,148]. A unique feature of the E channel is that its cation selectivity is dependent on the charge of the lipid environment, as the lipids form a part of the channel pore [149,150]. When reconstituted in membranes containing uncharged lipids, E protein is no longer selective toward cations [149]. As already discussed, alphavirus 6K has a preference for monovalent cations over divalent cations and anions [64,78].

### 3.4. Ion Channel Activity of 6K M2, Vpu, p7, and E Involved in Glycoprotein Trafficking, Particle Assembly, and Budding

During endosomal fusion, the M2 channel of IAV is activated under low-pH conditions, conducting protons from the acidic early endosomes into the virus particle for uncoating [16,151]. Once the particle enters the late endosomes, the M2 channel allows the influx of K^+^ ions that further prime the viral proteins for disassembly. Some reports indicate that M2 ion channel activity is not essential for entry, suggesting an alternate disassembly pathway [128,152]. Deleting the M2 ion channel domain does not affect viral replication in cell culture, and deleting the full-length protein delays growth kinetics [152]. The loss of M2 function has a more pronounced effect in mice than in cell culture, an observation also made in in vivo studies of alphavirus 6K and TF [35,37]. Upon genome replication and translation, the M2 protein traffics from the ER to the TGN, where it is again activated by low pH to conduct protons. This leads to the deacidification of the TGN, which prevents the premature maturation of the hemagglutinin (HA) glycoprotein [132]. M2 interferes with the glycosylation of HA and delays its trafficking through the Golgi compartment to the PM [14,153]. The M2 channel activates the host inflammasome pathway in the Golgi by disrupting ionic concentrations, releasing proinflammatory cytokines [1,154]. M2 mediates membrane scission at the neck of the budding virion independent of the endosomal sorting complex required for transport (ESCRT) machinery [8]. Thus, both M2 and 6K are involved in efficient glycoprotein trafficking and viral budding, although the exact mechanisms of 6K’s channel activity are not known.

The mechanism of Vpu channel-mediated viral release is not well understood. Only a small fraction of 6K and Vpu traffic to the PM, where these proteins are hypothesized to cause membrane depolarization, assisting in viral release [1,102,155]. Some functions have been identified for the Vpu TMD that promote release but are not directly related to its ion channel activity. For instance, the TM and cytosolic domains of Vpu degrade tetherin and the cluster of differentiation 4 (CD4) receptor to promote egress [1,24,156,157,158]. Mutating a conserved tryptophan residue at position 22 in the TMD of Vpu eliminates CD4 degradation [157]. Tetherin is a host protein that has been implicated in disrupting viral release, while CD4 is a known receptor for HIV-1 and can interact with the viral glycoprotein *env* in the ER to stall its trafficking to the cell surface [133,159].

HCV p7 is involved in glycoprotein processing, virus assembly, and the release of infectious virus particles but is not necessary for replication [1,10,134,151,160]. The ion channel activity of p7 enhances membrane permeability during infection—a feature common to the viroporin family [1,126]. p7 modulates the pH of the secretory pathway to make it conducive for the assembly and production of HCV particles [15,126]. p7 is also involved in the assembly of HCV core proteins into a capsid. Mutating the conserved dibasic residues in the cytoplasmic loop or the TM regions of p7 affects channel activity and leads to the accumulation of non-enveloped and incompletely assembled capsids [129,161]. p7, along with other non-structural proteins, is involved in the retrieval of HCV core proteins from lipid droplets, which act as storage units, to the sites of assembly [126]. The loss of p7 channel function hinders this retrieval, which in turn affects particle assembly, although the exact mechanism of this function is not clear [161]. Mutations in the dibasic residues can also lead to very low production of infectious particles and the loss of viability in vivo [160,162]. p7 does not seem to play a role in HCV entry as the deletion of p7 does not affect the specific infectivity of the virus [10,15]. However, one report suggests that p7 may be a virion component involved in entry as the channel inhibitor amantadine is able to block entry [163].

The SARS-CoV E TMD forms a cation-selective ion channel that is not regulated by voltage [150]. Introducing mutations in key residues of the channel domain, N15 and V25, abolishes the channel function in electrophysiology studies [150]. The N15A mutation attenuates the virus in vivo, leading to reduced mortality and disease manifestation in mice [34]. E protein can depolarize cellular membranes to induce membrane curvature, disrupt normal cellular function, and induce ER stress, leading to cell death and autophagy [1,3,36,164]. Similarly to E, SINV 6K, IAV M2, and HCV p7 can also induce apoptosis in cells by permeabilizing membranes and disrupting ion homeostasis [165]. The main function of the E protein in viral infection is related to the efficient assembly and release of virus particles via the secretory pathway. A recent study also suggests that E protein can help deacidify lysosomes and inactivate the lysosomal enzymes that would otherwise degrade the viral proteins, allowing the virus to use the lysosomal pathway for egress [166]. By disrupting calcium homeostasis, E protein can lead to the activation of host inflammasomes [147]. SARS-CoV-2 E protein can trigger inflammatory responses by upregulating cytokine expression and is associated with acute respiratory distress syndrome (ARDS), cytokine storm, and lung tissue damage during acute infection [36]. The activation of host inflammasomes by viroporin activity, as seen in the cases of M2 and E, could be one of the common mechanisms in respiratory infections that leads to lung tissue damage [167].

### 3.5. Interaction of 6K M2, Vpu, p7, and E with Viral and Host Proteins to Assist in Infection and Immune Evasion

Viroporins can assist in infection independent of their ion channel activity by interacting with host proteins through their cytoplasmic domains. Residues exposed in the ER lumen may also interact with proteins in the secretory pathway, but very little is known about these interactions for the viroporins discussed in this review.

**Figure 4:**
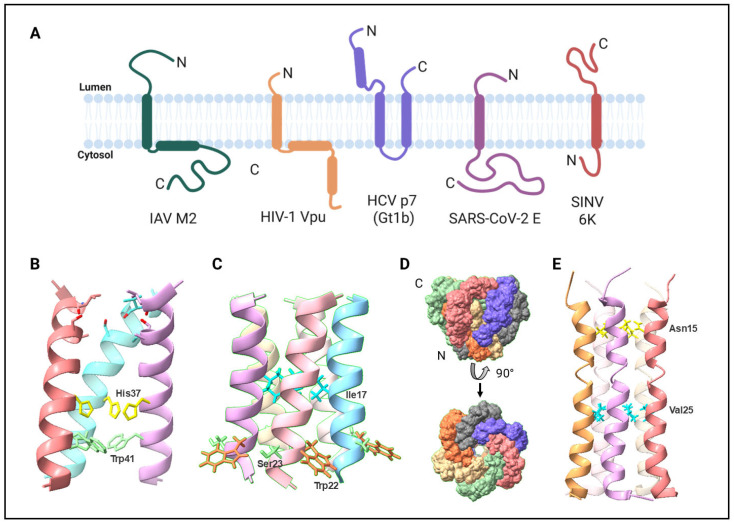
Monomeric membrane topology and oligomeric channel structures of viroporins. (**A**) Membrane topologies of monomeric IAV M2, HIV-1 Vpu, HCV p7 (Gt 1b), SARS-CoV-2 E, and SINV 6K (figure created with BioRender.com, https://www.biorender.com/, accessed on 1 June 2025). (**B**–**E**) Channel structures of (**B**) hexameric IAV M2 with the fourth chain hidden (PDB accession 3LBW) [197], (**C**) pentameric HIV-1 Vpu (PDB accession 1PI7) [198], (**D**) hexameric HCV p7 (Gt 5a) (PDB accession 2M6X) [199], and (**E**) pentameric SARS-CoV-2 E (PDB accession 7K3G) [200] proteins, showing important residues involved in channel function. Abbreviations: Gt, genotype; PDB, protein data bank. Structures were generated using UCSF ChimeraX software [201].

The cytoplasmic domain of IAV M2 contains an amphipathic helix at its N-terminus and a C-terminal tail (Figure 4A). The amphipathic helix is palmitoylated and contains the cholesterol recognition/interaction amino acid consensus (CRAC) motif, which allows M2 to bind to cholesterol [110,168]. This allows M2 to associate with the PM and assist in membrane scission and budding [1,8]. Through its C-terminal tail, M2 interacts with microtubule-associated protein 1 light chain 2 (LC3) and the viral protein matrix 1 (M1). LC3 is a host protein involved in the fusion of autophagosomes with lysosomes where protein cargo is degraded. M2 prevents this fusion from taking place and hampers autophagy to promote viral stability [168,169]. The interaction with M1 also allows M2 to support viral assembly and budding [1,170].

The cytoplasmic domain of HIV-1 Vpu contains two α-helices separated by a short loop and has sites for phosphorylation [171]. The first helix is involved in the interaction and degradation of CD4, and the second helix contains an EXXXLV motif needed for efficient tetherin degradation [172,173]. The phosphorylation of two highly conserved serine residues in the Vpu cytoplasmic domain also regulates the degradation of the CD4 receptor [133]. Thus, this domain allows Vpu to interfere with normal protein expression and trafficking in infected cells. Vpu plays a role in inducing cell death and evading immune responses through its cytoplasmic domain by interacting with and downregulating host immune factors [159,174]. Vpu is also involved in disrupting the function of the cellular K^+^ channel, TASK-1, which is involved in maintaining surface potential at the PM [133]. Vpu can oligomerize with TASK-1, due to structural homology, and inhibit its channel activity, leading to PM depolarization and viral release [175].

p7 functions as the signal sequence for the translocation of the non-structural protein NS2, which is involved in viral replication, and can bind to NS2 [176,177]. The interaction of p7 and NS2 is independent of p7’s ion channel activity and is critical for several downstream functions of both proteins that, in turn, affect assembly [126,177]. Together, p7 and NS2 recruit other HCV non-structural and core proteins to the sites of assembly [126,160]. p7 is also involved in immune evasion, but the exact mechanisms of most of p7’s functions during infection are not clear [178].

Coronavirus E protein has conserved cysteine residues just outside the TMD that are palmitoylated, affecting the subcellular localization and function of the protein [179]. A recent study has shown that the E protein of SARS-CoV-2 is a target for ubiquitination, which affects viral replication [180]. E protein can interact with the viral membrane protein (M) and assist in assembly [181,182]. The cytoplasmic domain of E contains a post-synaptic density protein-95 (PDZ)-binding motif that binds protein associated with Lin-Seven 1 (PALS1), a tight junction-associated protein, to disrupt the integrity of lung epithelial cells [183,184,185]. This motif also allows the E protein to bind to the host protein syntenin and activate the host inflammatory response [186]. Thus, E protein is a virulence factor that evades immune responses during infection. Recently, a PDZ-binding motif was also identified in CHIKV TF that allows TF to downregulate the human protein Scribble, a regulator of cell polarity [100]. This further supports the role of TF as a virulence factor involved in targeting host proteins during infection [101].

Other interacting partners for E protein are Bcl-xl, an anti-apoptotic host protein, and Toll-like receptor 2 (TLR-2). E protein binds Bcl-xl to prevent its function, thereby promoting apoptosis [187]. E protein is a ligand for Toll-like receptor 2 (TLR-2) and activates the NOD-like receptor (NLR) family pyrin domain-containing 3 (NLRP3) inflammasome pathway during infection [188,189]. However, a later report suggests that spike protein (S), and not E, is involved in the activation of the TLR-2 mediated NF-κB pathway [190]. Similarly to 6K and M2, E protein has a more striking effect on viral infection in vivo, affecting survival and immune response, but is not needed for viability in cell culture. The deletion of E in mice leads to better survival, reduced lung damage, the upregulation of stress responses, and the downregulation of proinflammatory cytokines [191,192,193]. The deletion of E also leads to an increase in macrophages and faster humoral and T cell responses during infection [191,193]. Like M2 and Vpu, SARS-CoV E can also influence the channel activity of cellular ion channels to facilitate viral infection [135,175,194,195,196].

### 3.6. Comparison of Structural Features of 6K, M2, Vpu, p7, and E

Based on their membrane topology and number of TM helices, Nieva JL et al. (2012) classified viroporins into two classes with two subclasses in each [1]. M2, Vpu, and E belong to Class I Subclass A and have a single membrane-spanning domain with the N-terminus in the ER lumen and the C-terminus in the cytosol (Figure 4A). While their TMDs are involved in channel formation, the cytoplasmic tails are accessible to host proteins for interactions and protein modifications. 6K and p7 have two TMDs, each with their N- and C-terminal ends in the ER lumen, and belong to Class II Subclass A [1], although, according to the new topology proposed for 6K, it would belong to Class I Subclass B [202].

X-ray crystal and NMR structures of the IAV M2 ion channel reconstituted in detergent micelles have been solved at different pH conditions, revealing a tetramer with binding sites for rimantadine and amantadine [197,203,204]. The M2 channel is activated in low-pH conditions when the histidine residues at position 37 in the pore are protonated and undergo a conformational change, allowing the channel to open (Figure 4B) [205]. The NMR structure of the TM helix of Vpu in detergent micelles has also been solved and modeled as a pentamer, the most stable oligomeric state, although Vpu can form larger oligomers as well [198,206,207,208]. The exact mechanism of Vpu channel activity is not well understood, but some key aromatic and hydrophobic residues, including the highly conserved serine at position 23, have been identified as important for channel function (Figure 4C) [209,210]. Vpu is also structurally similar to the host channel TASK-1 and can function in a voltage-dependent and -independent manner [133,210].

Several groups have solved NMR structures of p7 and modeled the protein as a hexameric or heptameric flower-shaped channel using different detergents [199,211,212,213]. The p7 channel is much larger relative to M2 and shows genotype-specific differences [214,215]. The channel is shaped like a funnel with highly conserved asparagine residues at position 9 of each monomer acting as a proton selectivity filter (Figure 4D) [214]. The two TM helices of p7 are separated by a short loop containing two highly conserved basic residues—K/R33 and R35—that are important for ion channel activity [216].

Recently, the structure of the pentameric SARS-CoV-2 E channel domain bound to the ion channel inhibitor HMA was solved using solid-state NMR spectroscopy in lipid bilayers mimicking the ERGIC (Figure 4E) [200]. In addition to their TMDs that form their respective ion channels, M2, Vpu, and E have extended cytoplasmic domains that interact with host proteins and mediate infection through channel-independent pathways. While the structure of alphavirus 6K has not been solved yet, Dey D. et al. (2020) proposed CHIKV 6K as a hexamer based on the size-exclusion chromatography profile of GST-tagged 6K protein [88].

## 4. Viroporin Inhibition and Therapeutic Potential for Pandemic Preparedness

Viroporins have been targeted for antiviral and vaccine development in the past [36,44,86,217,218,219,220]. Their importance in viral infections and their ability to functionally complement other viroporins, partially or fully, make them potential targets for broad-spectrum antivirals (Table 3). While sharing specific structural and functional similarities, viroporins also display significant diversity in sequence, oligomeric states, and virus-specific roles, as outlined in the previous section. For instance, although most viroporins are dispensable for infection, others such as SAV 6K are essential. And while most viroporins are incorporated into the virion, others such as HCV p7 are not. The context in which each viroporin must function can vary widely between viruses and their host organisms. These differences should be taken into account when designing and validating broad-spectrum antiviral candidates targeting this protein class.

Notwithstanding these differences, viruses seem to involve viroporins in basic aspects of pore formation and membrane remodeling. The development of common channel inhibitors not only will expand our current arsenal against clinically important viruses but has the potential to provide valuable insights into the function of newly identified and elusive viroporins. When coupled with functional complementation studies, they can reveal the following: (A) whether the chimeric viroporin is a functionally active ion channel; (B) the stage(s) of infection most affected by the loss of channel activity; and (C) the defects in maturation, particle assembly, and egress caused by the inhibition of viroporins. Some common channel inhibitors against 6K, Vpu, M2, p7, and E are discussed below.

### 4.1. Amantadine

The inhibition of M2 channel activity with amantadine and rimantadine greatly attenuates the virus in cell culture and mice [1,39,236]. In fact, amantadine became the first FDA-approved drug for the treatment of influenza infection back in the 1960s but was discontinued due to the development of drug resistance [237]. In the 1990s, the M2 channel was identified as its target, making M2 the first viroporin to be discovered and paving the way for subsequent viroporin research and targeted therapies [56,229]. The mutations that cause virus variants to become resistant have been explored previously and can be helpful in designing modified amantadine-like compounds targeting M2 in the future [214,238,239]. Structural studies have shown that amantadine and rimantadine can bind M2 at two sites: one inside the channel that directly occludes the channel pore; and the other outside the channel, which could allosterically cause the channel to close [40,203,204]. Chimeras of IAV and IBV variants expressing only the M2 channel show that the pore-binding site is the primary drug-binding site of amantadine and rimantadine [39,240,241]. Indeed, SHIV-Vpu and SINV-6K chimeras, with their channel domains replaced with that of M2, are also sensitive to rimantadine and amantadine [13,128].

Similarly to IAV M2, HCV p7, CHIKV 6K, and SARS-CoV/SARS-CoV-2 E proteins can be inhibited by amantadine [88,129,221,235,242]. While Vpu is not sensitive to amantadine, a single-amino-acid mutation in the HIV-1 Vpu channel sequence has been shown to make the virus sensitive to rimantadine [232,243]. Despite promising results in in vitro and cell culture-based inhibition assays, amantadine has not shown beneficial effects in clinical trials against HCV infection [222]. Amantadine/rimantadine sensitivity is also genotype-dependent, which may be explained by differences in sequences [211,214]. Amantadine binds to six equivalent sites in the hexameric p7 ion channel, with each molecule making contacts with hydrophobic residues of three p7 monomers [211]. The binding site is a hydrophobic pocket outside the channel cavity; thus, amantadine does not occlude the channel. Relative to M2, the p7 channel is too big to be blocked entirely by amantadine molecules. Amantadine, likely, has different mechanisms of inhibition in p7 and M2 [214]. Despite this, the hydrophobic nature and size of the binding sites and mutations that lead to resistance, by reducing the hydrophobicity of the binding sites, in p7 and M2 are similar [214]. Thus, common inhibitors can be developed against M2 and p7, even if the inhibition mechanism and the resultant effect on infection may be different for the viroporins. Amantadine is also an inhibitor of DNA viroporins such as the Kcv protein in plaque-forming chlorella virus, PBCV-1 [244].

### 4.2. HMA (Amiloride Derivatives)

HMA is known to inhibit HIV-1 infection by targeting the ion channel activity of Vpu [230,245,246,247]. HMA can also inhibit the HCV p7 channel in a sequence-dependent manner in in vitro and cell culture assays [15,145,211]. Wild-type SINV and an SINV-Vpu chimera were shown to be sensitive to HMA in a dose-dependent manner in a cell culture assay [13]. HMA, along with other amiloride derivatives, ethyl isopropyl amiloride (EIPA) and dimethyl amiloride (DMA), showed an antiviral effect against SARS-CoV-2 E protein channel activity during infection [38].

### 4.3. BIT225

BIT225 is a channel inhibitor and lead compound developed by the Australian company Biotron to target the ion channel activity of viroporins in HCV, HIV-1, and SARS-CoV-2 infections [218,219,220,233]. The compound is currently being tested in clinical trials and has shown antiviral effects in patients with HIV-1 and HCV in the past [248]. Since the pandemic, BIT225 has been tested against SARS-CoV-2 infections with promising results in mice [220].

## 5. Conclusions and Future Prospects

The lack of structural information for 6K and TF proteins has limited our understanding of their role in alphavirus infection. Future research that elucidates 6K’s structure, interaction with viral glycoproteins and host factors, and channel-specific function in infection will help uncover the mechanisms through which it promotes viral budding. Studies investigating TF’s role in infection in the absence of 6K are needed to understand TF-specific roles in infection and virus assembly. In particular, attempts should be made to determine whether TF interacts and co-traffics with the glycoproteins before being incorporated into the virus particle.

Viroporins are promising targets in the development of broad-spectrum antivirals and combination therapies. Comparative analysis of viroporin functions across distinct virus families can provide valuable insights for the identification and development of common antivirals. Such analysis should be followed up with experiments to evaluate viroporin complementation and inhibition not only in in vitro setups, but also in the context of in vivo infections where interactions with host and viral components may differ significantly. This approach can also lay the groundwork for discovering novel viroporin functions and mechanisms in elusive and newly identified viroporins.

## Figures and Tables

**Table 1:** Summary of 6K/TF deletion and mutation studies performed in various alphavirus/chimeric backgrounds with associated defects.

Deletion/Mutation	Virus		Phenotype	References
Deletion/mutation of palmitoylation sites in 6K	SINV	Sites in TF also affected	Decrease in virus yield Formation of multicored particles Deformation of budding virus particles Change in level of TF in virus particles	[61,71,79]
Deletion of 6K	SFV		Less efficient polyprotein cleavage Defect in budding and lower viral titer (host/cell-line-dependent) Reduced membrane fusion capacity in liposomal fusion assay No effect on glycoprotein production and heterodimerization No effect on specific infectivity and particle morphology	[32,81,82]
6K chimeras	SINV	SINV 6K replaced with RRV 6K	SINV (RR6K) chimera has lower titer (10% of WT) No effect on glycoprotein heterodimerization and trafficking	[83]
	SINV	SINV 6K-E1 replaced with RRV 6K-E1	SINV (RR6K-E1) has much lower titer (10^−7^ of WT) Defects in glycoprotein heterodimerization and interaction with nucleocapsids No effect on glycoprotein processing and trafficking Revertants have mutations in E2 that improve virus yield	[83,84]
	SINV	6K channel replaced with M2, Vpu, and p7	Defects of 6K deletion rescued partially in M2, Vpu, and p7 chimeras	[13]
Deletion of 6K and TF	SINV	Residues 24–45 deleted in 6K (no TF produced)	2 log reduction in viral titer Defects in glycoprotein processing and trafficking 6K Q21L (∆24–45) revertant rescues processing defect (by enhancing membrane association and resolving signalase cleavage defect) but does not affect budding 6K protein co-expression in trans does not rescue 6K mutation/deletion-associated defects	[80,85]
Mutations in 6K interfacial domains	SINV	^9^(YLW→AAA)^11^ and ^18^(FWV→AAA)^20^	Lower membrane permeabilization and toxicity in *E. coli* and BHK cells	[63]
Deletion of TF	SFV	6K unaffected	~56% reduction in growth relative to WT SFV (plaque assay in BHK cells)	[67]
Deletion of TF	SINV	6K unaffected	Budding defect and reduction in viral titer No effect on genome synthesis and specific infectivity No effect on expression and fusogenicity of glycoproteins Reduced mortality in mouse model	[35]
Deletion of 6K	CHIKV		6K deletion mutant protects against high dose of CHIKV after single immunization and elicits humoral and cellular responses	[86]
Deletion of 6K	SAV		Loss of viability Viral proteins translated but do not reach PM for budding	[73]
Deletion of 6K and TF	RRV	No effect of in-frame deletion on polyprotein processing	Mutant virus defective in virion release but not particle production in BHK-21 cells RRV(∆6K) more sensitive to pH and temperature changes than WT RRV Lower viral titers and milder disease outcomes in mice compared to WT RRV Mice immunized with RRV(∆6K) show faster viral elimination upon secondary infection with WT RRV	[37]
Deletion of 6K and TF	SINV	Except signal peptide	Approximately 4 log reduction in titer Reduced E2 surface expression at 6 and 12hpi (rescued by HIV-1 Vpu) Impaired CPV-II formation	[13]
Deletion of 6K	GETV (Getah virus)		1–2 log reduction in viral titer 24hpi Increase in number of cell-associated viruses Hampered E2 glycoprotein trafficking to PM Decreased thermostability at 52 °C and 56 °C Milder disease outcomes in vivo No effect on viral entry	[87]

Abbreviations: WT, wild type; BHK cells, baby hamster kidney cells; hpi, hours post infection.

**Table 2:** List of TF-specific properties and functions in alphavirus infection.

Properties of TF	Virus/Cell System	Details	References
−1 PRF	Multiple viruses	TF is a product of −1 PRF in alphavirus 6K sequences	[66,67,77]
RNA secondary structure	Multiple viruses	Species-specific diversity exists in RNA secondary structures downstream of the PRF slip site	[77]
Palmitoylation	SINV	TF palmitoylation affects its subcellular localization and incorporation into the budding virion	[71,99]
Oligomerization	Ectopic expression of CHIKV TF in 293T cells	TF cysteine residues are involved in oligomerization TF palmitoylation reduces its oligomerization	[95]
Ion conductance	Maltose-binding protein-tagged CHIKV TF incorporated in lipid bilayer	CHIKV TF conducts ions in lipid bilayer membranes in vitro	[89]
Effect on assembly and budding	SINV, SFV	TF affects viral budding and particle morphology	[35,67]
Effect on entry and fusion		NA	
Protein–protein interactions	Ectopic expression of CHIKV proteins in 293T cells	TF can interact with most CHIKV proteins except E3 TF interacts with nsp1 and nsp3 in a palmitoylation-dependent manner Potential interaction with human Scribble protein	[95,100]
Effect on IFN response	SINV	TF antagonizes IFN response Mechanism unknown	[101]
Effect on virulence in vivo	SINV	TF promotes infection in mice	[35]

Abbreviations: PRF, programmed ribosomal frameshifting.

**Table 3:** Inhibitor studies against viroporins—common channel inhibitors.

Viroporin	Length	Membrane-Spanning Domains	Oligomeric State	Inhibitor	References
6K	55aa (SINV)	1–2 TMD	Unknown	HMA	[13]
	61aa (CHIKV)			Amantadine	[88]
p7	63aa	2TMD	Hexamer	Amantadine/ Adamantanes	[15,42,163,221,222,223]
				HMA	[15,145,163,211]
				Long acyl-chain iminosugar derivatives	[43,224,225,226]
				BIT225	[218]
M2	97aa	1TMD	Tetramer	Amantadine	[40,56,204,227,228,229]
Vpu	81aa	1TMD	Pentamer	Amiloride derivatives (HMA)	[230,231]
				Rimantadine	[232]
				BIT225	[233]
SARS-CoV and SARS-CoV-2 E	75–76aa	1TMD	Pentamer	Amiloride derivatives (HMA, EIPA, DMA)	[38,234]
				Amantadine	[235]
				BIT225	[220]
				BE-33	[36]

Abbreviations: BIT225, N-(5-(1-methyl-1H-pyrazol-4-yl)-napthalene-2-carbonyl)-guanidine; BE-33, modified Berbamine (see reference for details); TMD, transmembrane domain.
